# Supplementary material for: Restoration of lysosomal acidification rescues autophagy and metabolic dysfunction in non-alcoholic fatty liver disease
Source: Nat Commun. 2023 May 4;14:2573. doi: 10.1038/s41467-023-38165-6 (PMC10160018; doi:10.1038/s41467-023-38165-6)
Supplement: Supplementary file 1 — Supplementary Information [file 41467_2023_38165_MOESM1_ESM.pdf]

Supplementary Information

Restoration of Lysosomal Acidification Rescues Autophagy and Metabolic Dysfunction in Non-alcoholic Fatty Liver Disease

Jialiu Zeng[1,2]*, Rebeca Acin-Perez[3,†], Essam A. Assali[3,†], Andrew Martin[1], Alexandra J. Brownstein[3], Anton Petcherski[3], Lucía Fernández del Rio[3], Ruiqing Xiao[4,5], Chih Hung Lo[2], Michaël Shum[3,6], Marc Liesa[3,6,7,8], Xue Han[1], Orian S. Shirihai[3,6,9,]* and Mark W. Grinstaff[1,4,9,]*

[1]Department of Biomedical Engineering, Boston University, Boston, MA 02215.

[2]Lee Kong Chian School of Medicine, Nanyang Technological University, Singapore, Singapore 308232.

[3]Division of Endocrinology, Department of Medicine, David Geffen School of Medicine, University of California, Los Angeles, Los Angeles, CA 90045.

[4]Department of Chemistry, Boston University, Boston, MA 02215.

[5]Shenzhen Middle School, Shenzhen, Guangdong 518001, China

[6]Molecular and Medical Pharmacology, David Geffen School of Medicine at University of California, Los Angeles, Los Angeles, CA 90045.

[7]Molecular Biology Institute at University of California, Los Angeles, Los Angeles, CA 90095.

[8]Institut de Biologia Molecular de Barcelona, IBMB, CSIC, Baldiri Reixac 4-8, Barcelona, Catalonia, 08028, Spain.

[9]Department of Medicine, Boston University School of Medicine, Boston, MA 02118.

*To whom correspondence should be addressed: jialiu.zeng@ntu.edu.sg; OShirihai@mednet.ucla.edu; mgrin@bu.edu.

† Equal contributions

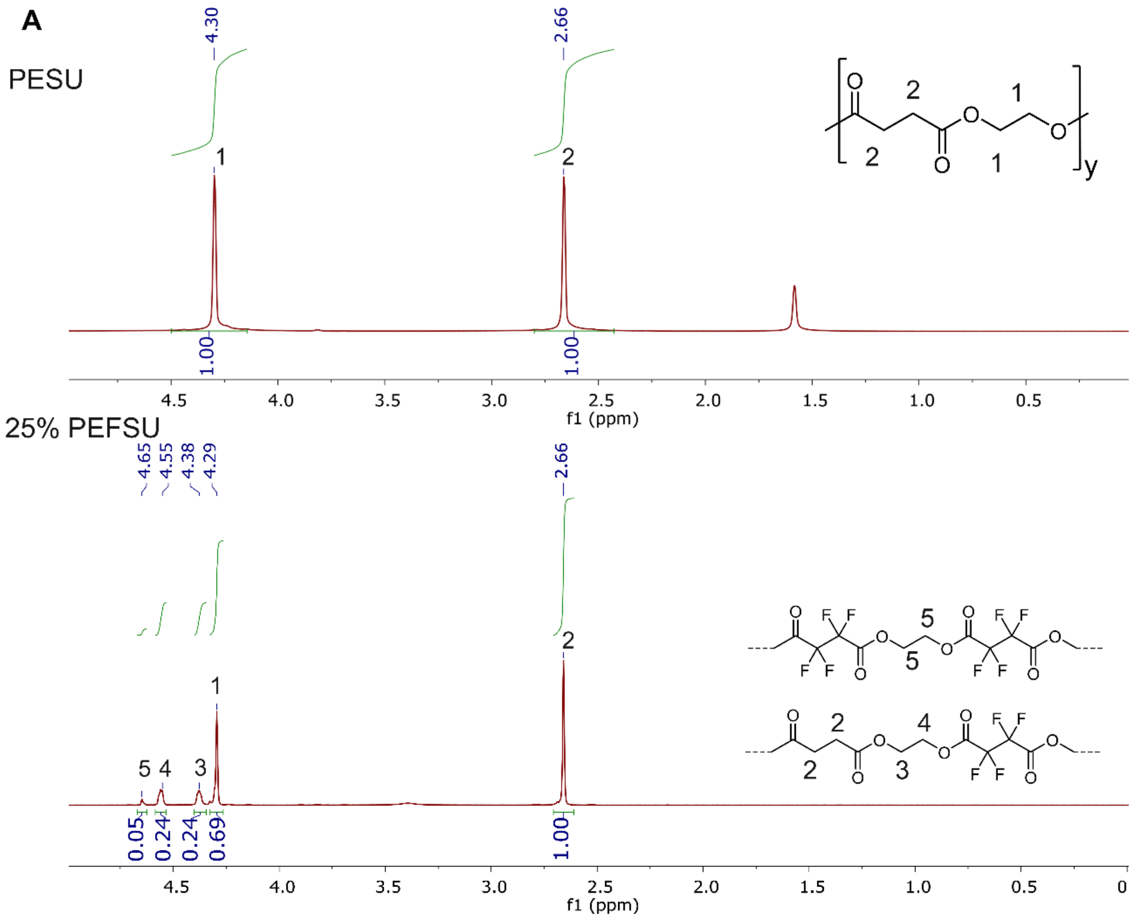
A
PESU
4.30
2.66
1
2
1.00
1.00
4.5
4.0
3.5
3.0
2.5
2.0
1.5
1.0
0.5
f1 (ppm)
25% PEFSU
4.65
4.55
4.38
4.29
2.66
5 4
3
1
2
0.05
0.24
0.24
0.69
1.00
0
f1 (ppm)

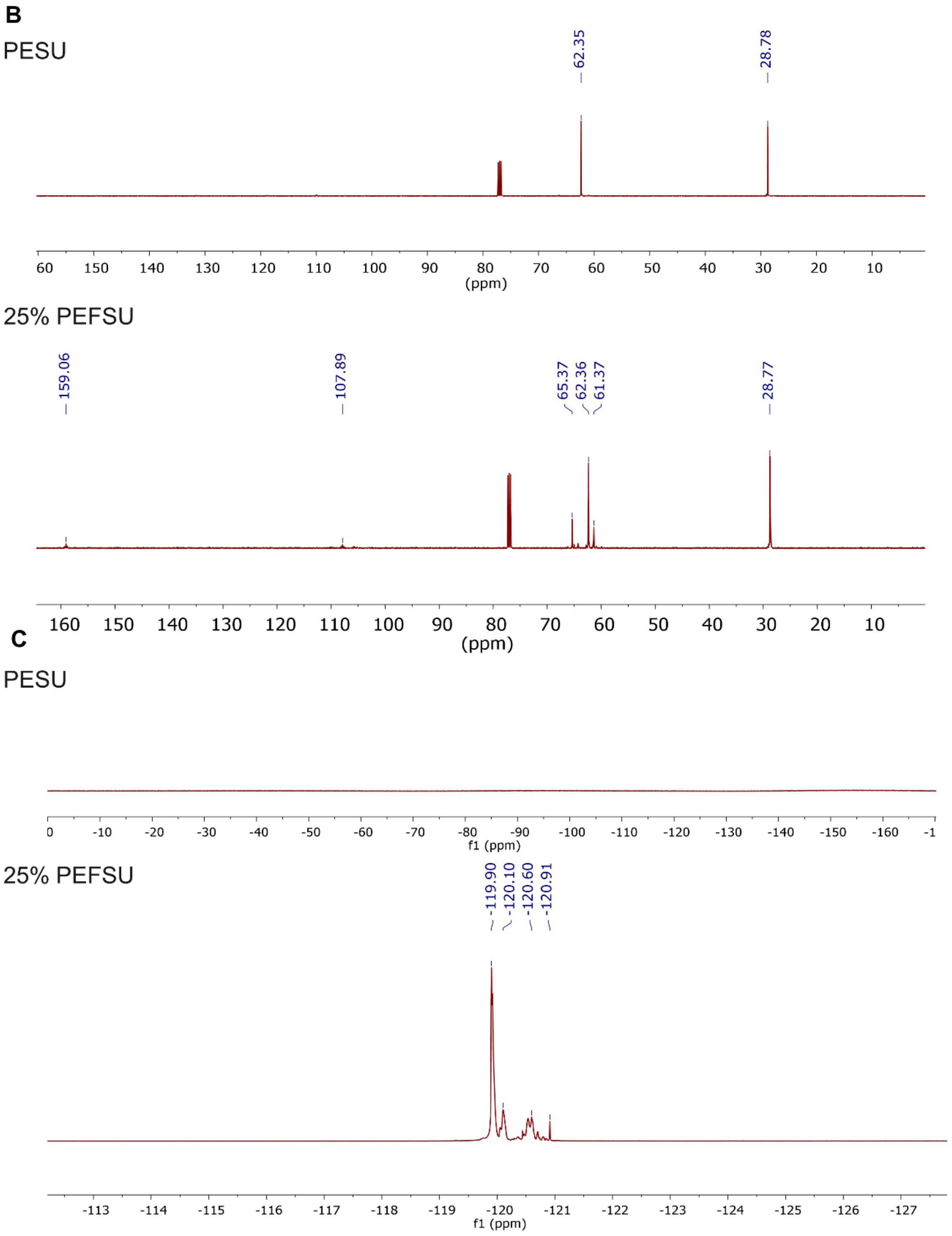

Supplementary Figure. 1.

Material characterizations for PESU and 25% PEFSU polymer. (A) ^{1}H NMR spectra of PESU and 25% PEFSU. ^{1}H NMR [(500 MHz, CDCl3): PESU δ 4.30 (s, 1H), 2.66 (s, 1H)], [25% PEFSU δ 2.66 (s, 1H), 4.29 (s, 0.69H), 4.38 (s, 0.24H), 4.55 (s, 0.24H), 4.65 (s, 0.05H)]. (B) ^{13}C NMR spectra of PESU and 25% PEFSU. ^{13}C NMR [(500 MHz, CDCl3): PESU, 62.35, 28.78], [25%

PEFSU 159.06, 107.89, 62.36, 61.37, 28.77]. (C) ^{19}F NMR spectra of 25% PEFSU. ^{19}F NMR [(500 MHz, CDCl3): 25 % PEFSU -120.91, -120.60, -120.10, -119.90].

A

	PESU	25% PEFSU
$\overline{M_n}$	9740	10082
$\overline{M_w}$	11104	12098

B

	Control NPs	acNPs
Size (nm)	105.1 ± 5.8	96.6 ± 4.3
Zeta Potential, ζ (mV)	-30.35 ± 3.5	-25.36 ± 2.9

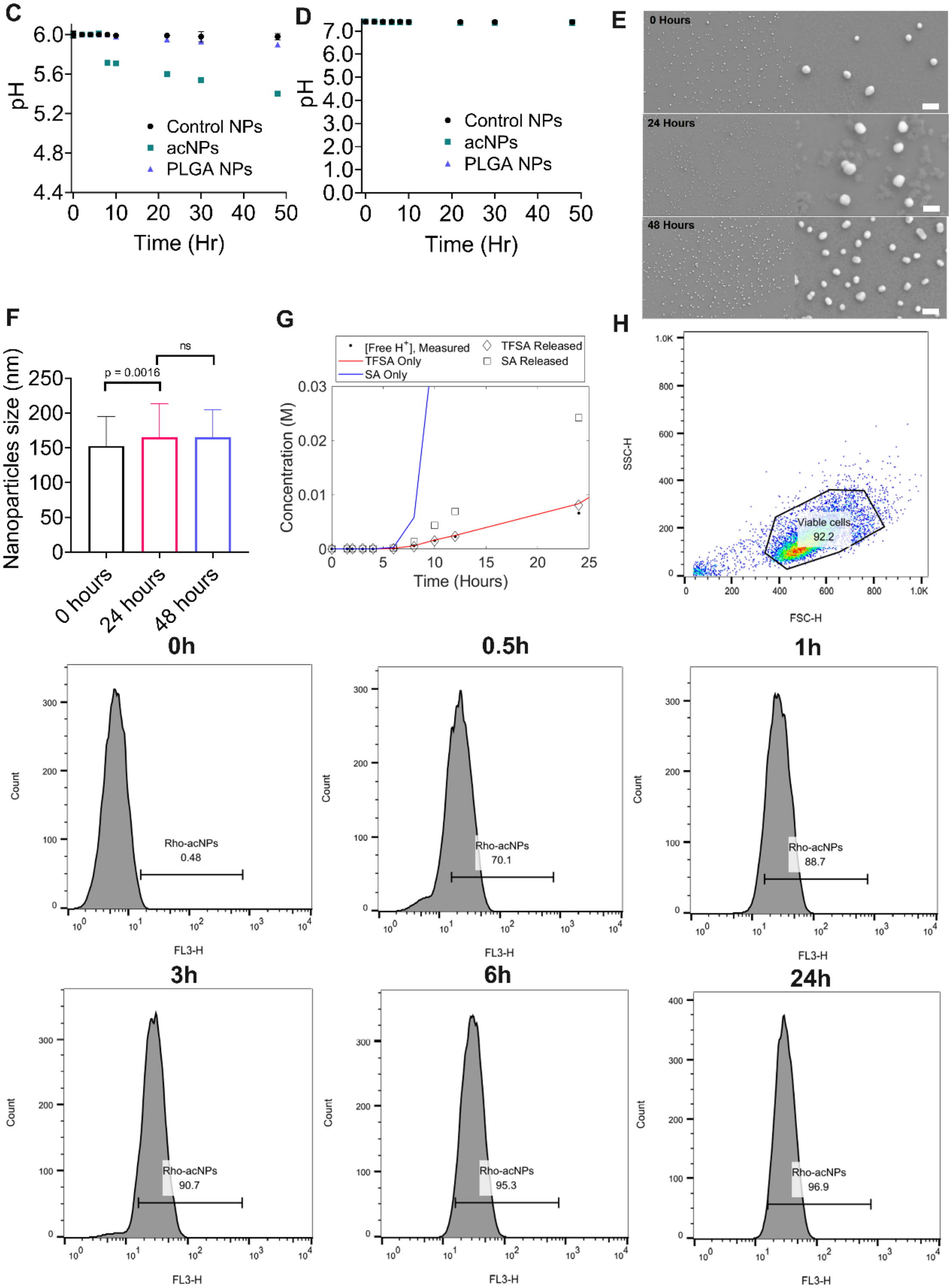

Supplementary Figure. 2.

(A) Molecular weights of PESU and 25% PEFSU polymer. (B) Size and zeta potential of control NPs and acNPs determined using dynamic light scattering (DLS) and zeta potentializer. (C) Degradation curve of control NPs, acNPs and PLGA NPs in 20 mM pH 6.0 (N = 3 independent experiments) and (D) pH 7.4 buffered solution. PLGA NPs did not significantly acidify either pH 6.0 or pH 7.4 buffer compared to acNPs (N = 3 independent experiments). (E) SEM micrographs of acidic NPs (25% PEFSU polymer) degradation over 48 hours. (F) Quantification of nanoparticle sizes using ImageJ (n=200 nanoparticles analyzed per condition). (G) We used the measured free acid in solution (based on pH measured in DI water) (black filled dots) to calculate the amount of TFSA (black diamonds) and SA (black squares) in solution to produce these pH changes assuming released acid follows the known polymer composition of 25% TFSA, 75% SA (generated from 25% PEFSU degradation). We then generated theoretical bounds for TFSA and SA combinations in solution by calculating the amount of TFSA only (red line) or SA only (blue line) to also yield these pH changes from their respective homopolymers. A large concentration of SA only is needed to generate significant changes in pH (PESU polymer), while it requires relatively little TFSA (< 0.005 M) release from 25% PEFSU to induce these changes. The equations used for these calculations is in Supplementary Note. (H) Representative images of gating strategy used for flow cytometry analysis of Rho-acNPs uptake in cells. FL3-H indicates the red fluorescence channel on 620 FACscan. Two-tailed unpaired t test (F); data are expressed as means ± SD. n.s. not statistically significant. Source data are provided as a Source Data file.

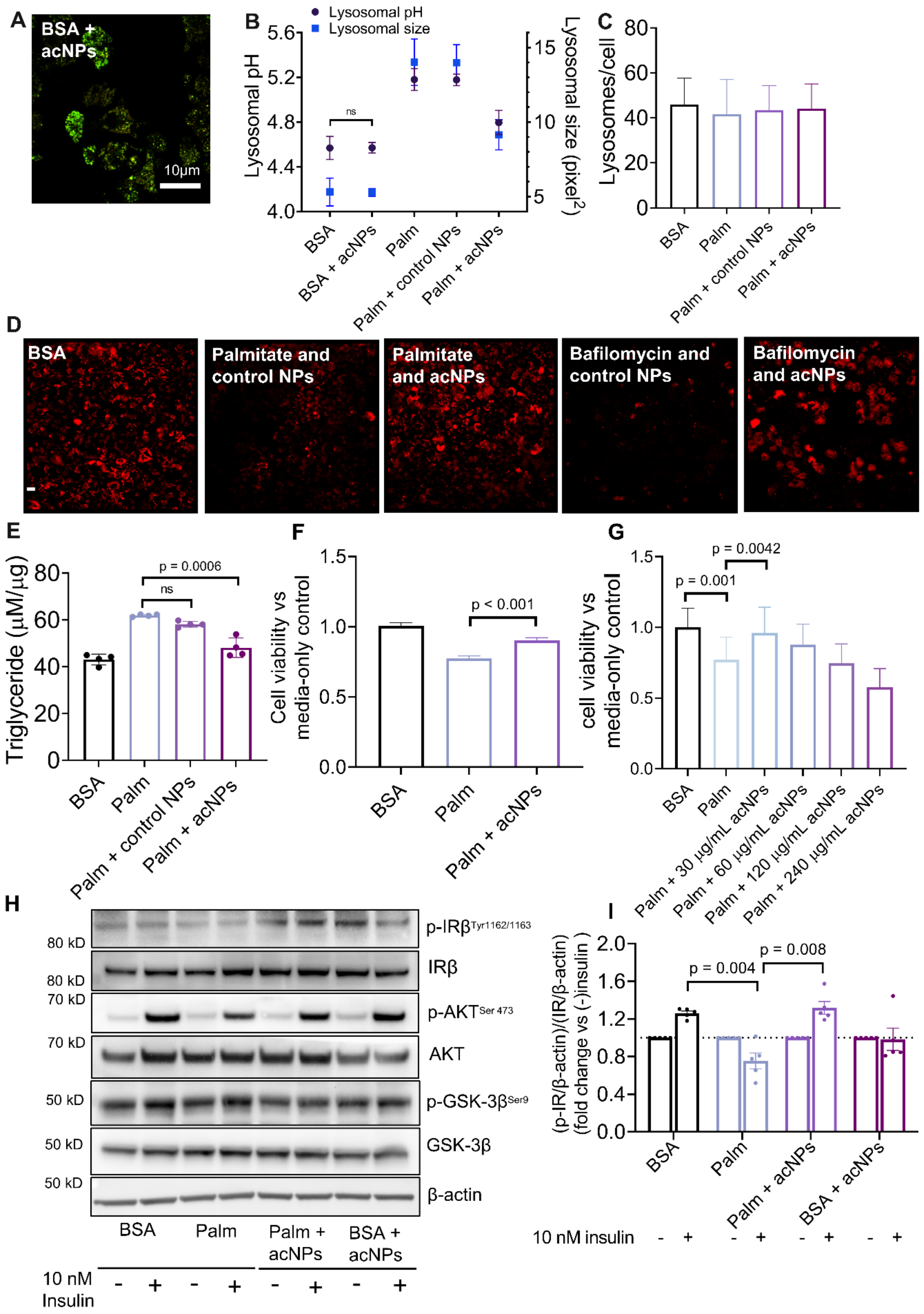

A
BSA + acNPs
10μm
B
Lysosomal pH
Lysosomal size
Lysosomal pH
Lysosomal size (pixel²)
ns
BSA
BSA + acNPs
Palm
Palm + control NPs
Palm + acNPs
C
Lysosomes/cell
BSA
Palm
Palm + control NPs
Palm + acNPs
D
BSA
Palmitate and control NPs
Palmitate and acNPs
Bafilomycin and control NPs
Bafilomycin and acNPs
E
Triglyceride (μM/μg)
p = 0.0006
ns
BSA
Palm
Palm + control NPs
Palm + acNPs
F
Cell viability vs media-only control
p < 0.001
BSA
Palm
Palm + acNPs
G
cell viability vs media-only control
p = 0.0042
p = 0.001
BSA
Palm
Palm + 30 μg/mL acNPs
Palm + 60 μg/mL acNPs
Palm + 120 μg/mL acNPs
Palm + 240 μg/mL acNPs
H
80 kD
80 kD
70 kD
70 kD
50 kD
50 kD
50 kD
p-IRβ Tyr1162/1163
IRβ
p-AKT Ser 473
AKT
p-GSK-3β Ser9
GSK-3β
β-actin
BSA
Palm
Palm + acNPs
BSA + acNPs
10 nM Insulin
I
(p-IR/β-actin)/(IR/β-actin) (fold change vs (-)insulin)
p = 0.004
p = 0.008
BSA
Palm
Palm + acNPs
BSA + acNPs
10 nM insulin

J

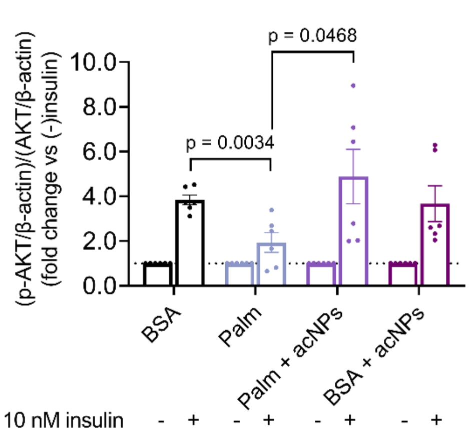

K

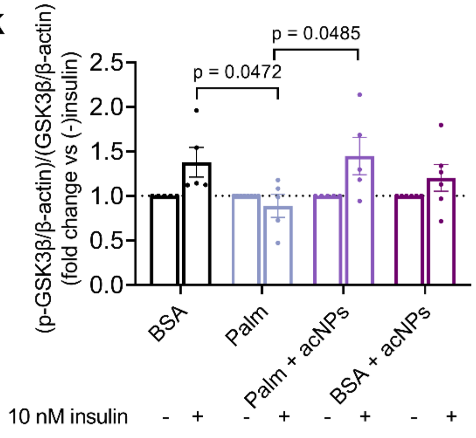

Supplementary Figure. 3.

acNPs function on lysosomal number, cathepsin L activity and autophagic flux in HepG2 cells. (A) Lysosensor yellow/blue imaging of BSA treated with acNPs. (B) Quantification of BSA and acNPs treated lysosomal pH and size (N=3 independent experiments with n=20 cells per condition). (C) Lysosome number per cell quantified from different treatment conditions (N=3 independent experiments with n=20 cells per condition). (D) Magic red imaging of lysosomes with different conditions, puncta indicate lysosomes. Palmitate treatment reduces lysosomal cathepsin L enzyme activity, hence reducing overall fluorescence. Addition of acNPs increases fluorescent puncta indicating restoration of lysosome cathepsin L activity. When bafilomycin, a lysosome V-ATPase inhibitor is added, lysosomal cathepsin L activity is reduced due to reduced lysosomal acidity. Treatment with acNPs acidifies lysosomes and restore lysosomal cathepsin L activity. Bar = 20 µm. (E) HepG2 cells triglyceride levels under different treatment conditions (N=4 independent experiments). (F) Cell viability of HepG2 cells (N=3 independent experiments, n=20 cells per condition), and (G) Primary human hepatocytes under palmitate conditions, and palmitate treated with nanoparticles (N=3 independent experiments). (H – K) Representative western blot exposure images showing protein expression data for insulin signaling pathway proteins. IR: Insulin receptor β (4B8); p-IR: phosphor-insulin receptor (Tyr 1162 and 1163); AKT: protein kinase B; p-AKT: phospho-protein kinase B (Ser 473); GSK3β: glycogen synthase kinase-3 beta; p-GSK3β: phospho-glycogen synthase kinase-3 beta (Ser 9). All results are expressed as a fold increase in the insulin-stimulated state relative to the unstimulated state (Palm = Palmitate, N=5 independent experiments). Two-tailed unpaired t test (B, E, F, G, I, J, K); data are expressed as means ± SD. n.s. not statistically significant. Source data are provided as a Source Data file.

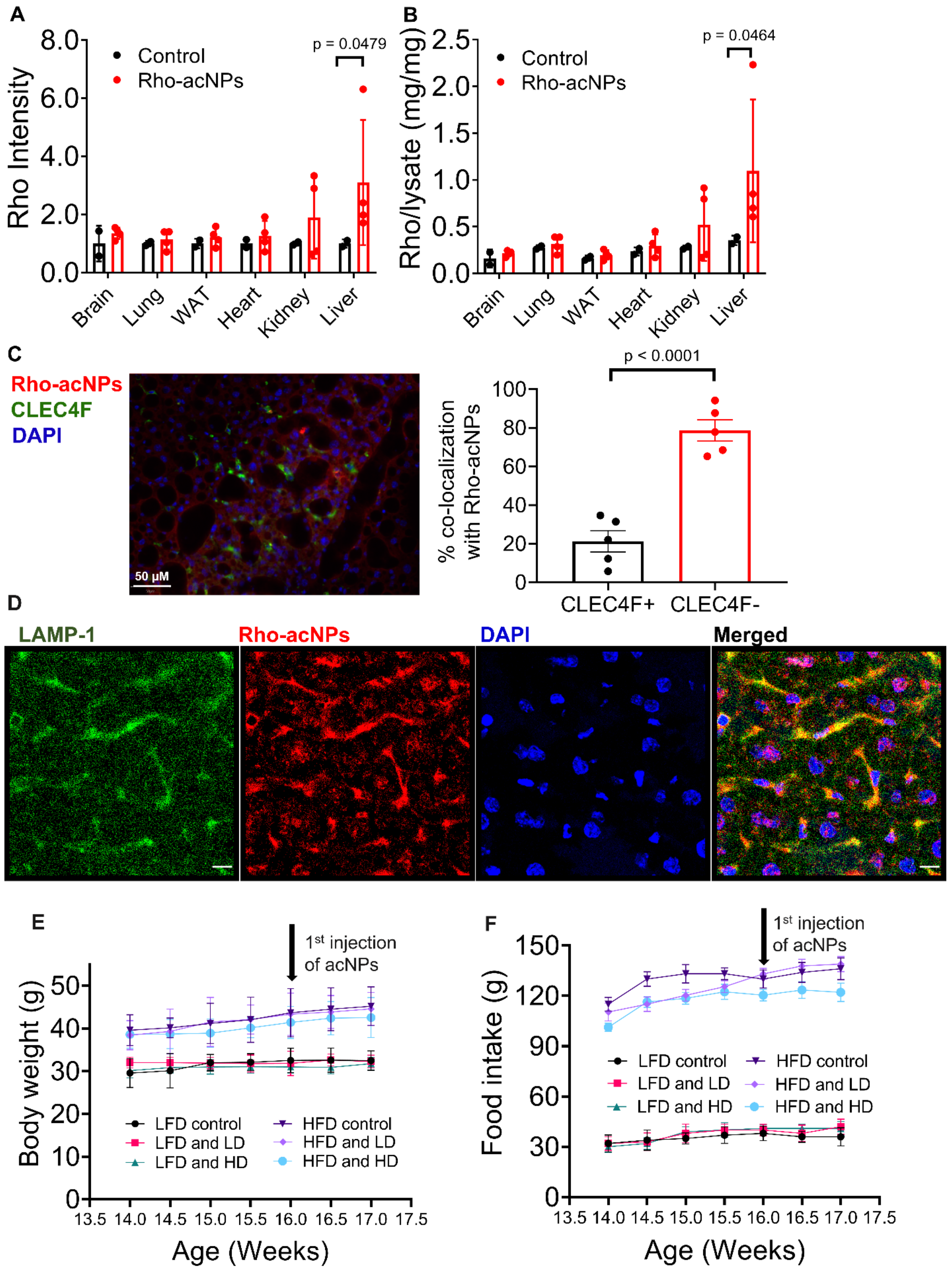

A
Rho Intensity
Control
Rho-acNPs
p = 0.0479
Brain
Lung
WAT
Heart
Kidney
Liver
B
Rho/lysate (mg/mg)
Control
Rho-acNPs
p = 0.0464
Brain
Lung
WAT
Heart
Kidney
Liver
C
Rho-acNPs
CLEC4F
DAPI
50 μM
p < 0.0001
% co-localization with Rho-acNPs
CLEC4F+
CLEC4F-
D
LAMP-1
Rho-acNPs
DAPI
Merged
E
1st injection of acNPs
Body weight (g)
Age (Weeks)
LFD control
LFD and LD
LFD and HD
HFD control
HFD and LD
HFD and HD
F
1st injection of acNPs
Food intake (g)
Age (Weeks)
LFD control
LFD and LD
LFD and HD
HFD control
HFD and LD
HFD and HD

Supplementary Figure. 4.

acNPs biodistribution and safety in mice. (A – B) Biodistribution of Rho-acNPs injection in mice. acNPs show significant accumulation in liver tissues (n=2 animals for control group, n=4 animals for Rho-acNPs group). (C) Representative confocal images showing that Rho-acNPs colocalize mainly in hepatocytes and not Kupffer cells, which are stained with anti-CLEC4F (n=5 animals for treatment group). Bar = 50 µm. (D) Rho-acNPs co-localize in the lysosomes of liver hepatocytes (n= 3 animals per condition). Bar = 10 µm.. (E) Body weight change and (F) food intake for mice across the treatment period. The acNPs treatment did not result in significant weight loss or decrease in food intake. Two-tailed unpaired t test (A, B, C); data are expressed as means ± SD. Source data are provided as a Source Data file.

Single injection of acNPs					
**Parameter**		**HFD Control**	**HFD and LD**	**HFD and HD**	**Reference values**
WBC	K/uL	9.64 ± 2.4	9.33 ± 1.4	11.6 ± 2.3	1. 8 - 10.7
NE	K/uL	2.40 ± 1.1	2.74 ± 0.5	2.63 ± 0.45	0.1 - 2.4
LY	K/uL	6.15 ± 1.7	6.21 ± 1.2	8.53 ± 1.7	0.9 - 9.3
MO	K/uL	0.41 ± 0.10	0.25 ± 0.10	0.32 ± 0.12	0.0 - 0.4
EO	K/uL	0.54 ± 0.05	0.10 ± 0.04	0.08 ± 0.02	0.0 – 0.2
BA	K/uL	0.14 ± 0.02	0.03 ± 0.01	0.03 ± 0.01	0.0 - 0.2
NE	%	22.9 ± 5.3	29.6 ± 4.1	22.8 ± 3.2	6.6 – 38.9
LY	%	66.4 ± 7.0	66.4 ± 3.9	73.6 ± 8.1	55.8 – 91.6
MO	%	4.7 ± 0.9	2.8 ± 1.2	2.6 ± 0.3	0.0 – 7.5
EO	%	0.47 ± 0.04	0.95 ± 0.13	0.71 ± 0.05	0.0 – 3.9
BA	%	1.28 ± 0.02	0.29 ± 0.01	0.25 ± 0.01	0.0 – 2.0
RBC	M/uL	10.1 ± 1.6	14.1 ± 0.9	10.2 ± 1.9	6.36 - 9.42
Hb	g/dL	14.3 ± 2.4	9.76 ± 0.7	14.6 ± 2.3	11.0 - 15.1
HCT	%	44.9 ± 8.0	42.6 ± 4.1	44.1 ± 7.9	35.1 - 45.4
MCV	fL	44.3 ± 2.1	43.6 ± 1.4	43.1 ± 0.7	45.4 - 60.3
MCH	pg	14.3 ± 0.8	14.4 ± 0.1	14.4 ± 0.5	14.1 – 19.3
MCHC	g/dL	32.3 ± 2.8	33.1 ± 1.1	33.3 ± 1.7	30.2 - 34.2
RDW	%	19.1 ± 0.7	18.1 ± 0.7	17.9 ± 0.8	12.4 - 27.0
PLT	K/uL	355 ± 115	788 ± 90	629 ± 142	592 - 2972
MPV	fL	5.93 ± 0.3	5.50 ± 0.2	5.90 ± 0.1	5.0 - 20.0

Supplementary Table. 1.

Hematological parameters analyzed for mice treated with either low dose or high dose acNPs for with a single injection. There is no significant change across all the hematological parameters for either low dose or high dose acNPs (n = 4 animals per condition). Data are expressed as means ± SD.

Multiple injections of acNPs					
Parameter		HFD Control	HFD and LD	HFD and HD	Reference values
WBC	K/uL	12.3 ± 1.0	9.78 ± 2.3	8.35 ± 2.0	1. 8 - 10.7
NE	K/uL	1.95 ± 0.5	3.05 ± 0.8	2.79 ± 1.1	0.1 - 2.4
LY	K/uL	9.26 ± 2.7	6.15 ± 2.1	5.35 ± 2.0	0.9 - 9.3
MO	K/uL	0.72 ± 0.08	0.36 ± 0.10	0.11 ± 0.05	0.0 - 0.4
EO	K/uL	0.26 ± 0.05	0.16 ± 0.03	0.08 ± 0.01	0.0 – 0.2
BA	K/uL	0.11 ± 0.02	0.07 ± 0.01	0.01 ± 0.0	0.0 - 0.2
NE	%	15.9 ± 2.3	32.6 ± 4.5	36.6 ± 6.2	6.6 – 38.9
LY	%	75.3 ± 5.9	61.7 ± 4.6	60.9 ± 5.5	55.8 – 91.6
MO	%	5.8 ± 1.2	3.6 ± 1.3	1.4 ± 0.2	0.0 – 7.5
EO	%	2.1 ± 0.2	1.5 ± 0.3	0.95 ± 0.06	0.0 – 3.9
BA	%	0.87 ± 0.05	0.69 ± 0.03	0.16 ± 0.01	0.0 – 2.0
RBC	M/uL	7.78 ± 1.3	9.15 ± 1.2	9.51 ± 0.8	6.36 - 9.42
Hb	g/dL	14.4 ± 0.4	14.1 ± 0.8	12.9 ± 1.1	11.0 - 15.1
HCT	%	46.6 ± 4.9	42.8 ± 5.5	40.7 ± 3.4	35.1 - 45.4
MCV	fL	59.9 ± 5.0	47.5 ± 8.0	42.8 ± 0.5	45.4 - 60.3
MCH	pg	18.5 ± 1.8	15.6 ± 1.7	13.6 ± 0.1	14.1 – 19.3
MCHC	g/dL	30.9 ± 2.5	33.2 ± 3.5	31.7 ± 0.6	30.2 - 34.2
RDW	%	30.0 ± 4.1	22.2 ± 4.2	19.8 ± 0.8	12.4 - 27.0
PLT	K/uL	2125 ± 290	1033 ± 160	947 ± 107	592 - 2972
MPV	fL	5.8 ± 0.9	6.16 ± 0.8	5.88 ± 0.4	5.0 - 20.0

Supplementary Table. 2.

Hematological parameters analyzed for mice treated with either low dose or high dose acNPs for multiple injections (three injections) over a period of six days. There is no significant change across all the hematological parameters for either low dose or high dose acNPs (n = 4 animals per condition). Data are expressed as means ± SD.

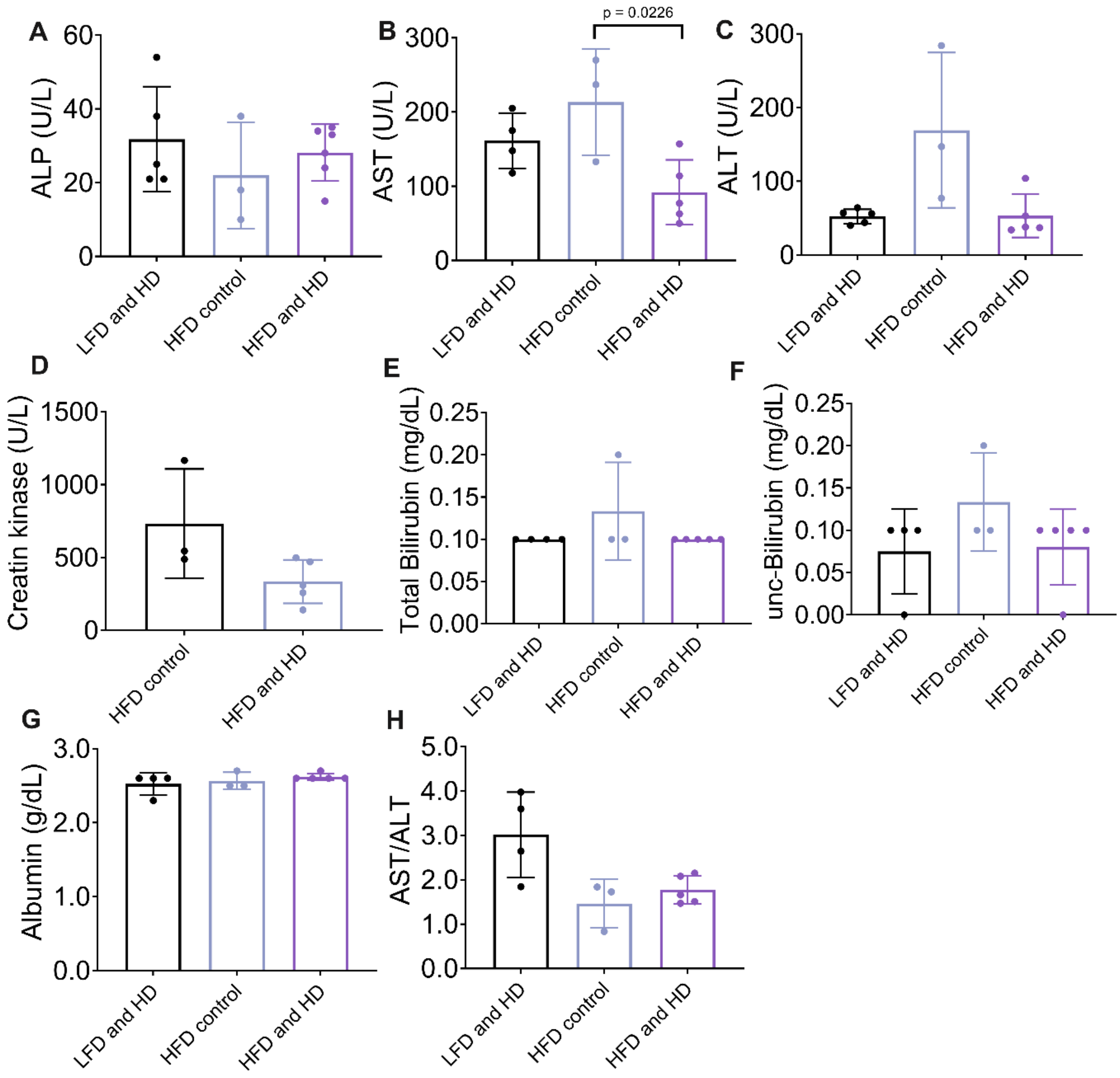

Supplementary Figure. 5.

Serum indicators in C57BL/6N mice treated with acNPs. Serum (A) Alkaline Phosphatase (ALP), (B) Aspartate transaminase (AST). (C) Alanine transaminase (ALT) (For A – C, n=4 animals for LFD and HD group, n=3 animals for HFD control group, n=6 animals for HFD and HD group). (D) Creatine kinase (n=3 animals for HFD control, n=5 animals for HFD and HD), (E) total bilirubin, (F) unconjugated bilirubin, (G) Albumin, and (H) AST/ALT ratio were analyzed. (For E – H, n=4 animals for LFD and HD, n=3 animals for HFD control, n=5 animals for HFD and HD). acNPs treatment reduced ALP, AST, ALT, creatine kinase, total bilirubin, unconjugated bilirubin levels, indicating improvement in liver function. Two-tailed unpaired t test (B); data are expressed as means ± SD. Source data are provided as a Source Data file.

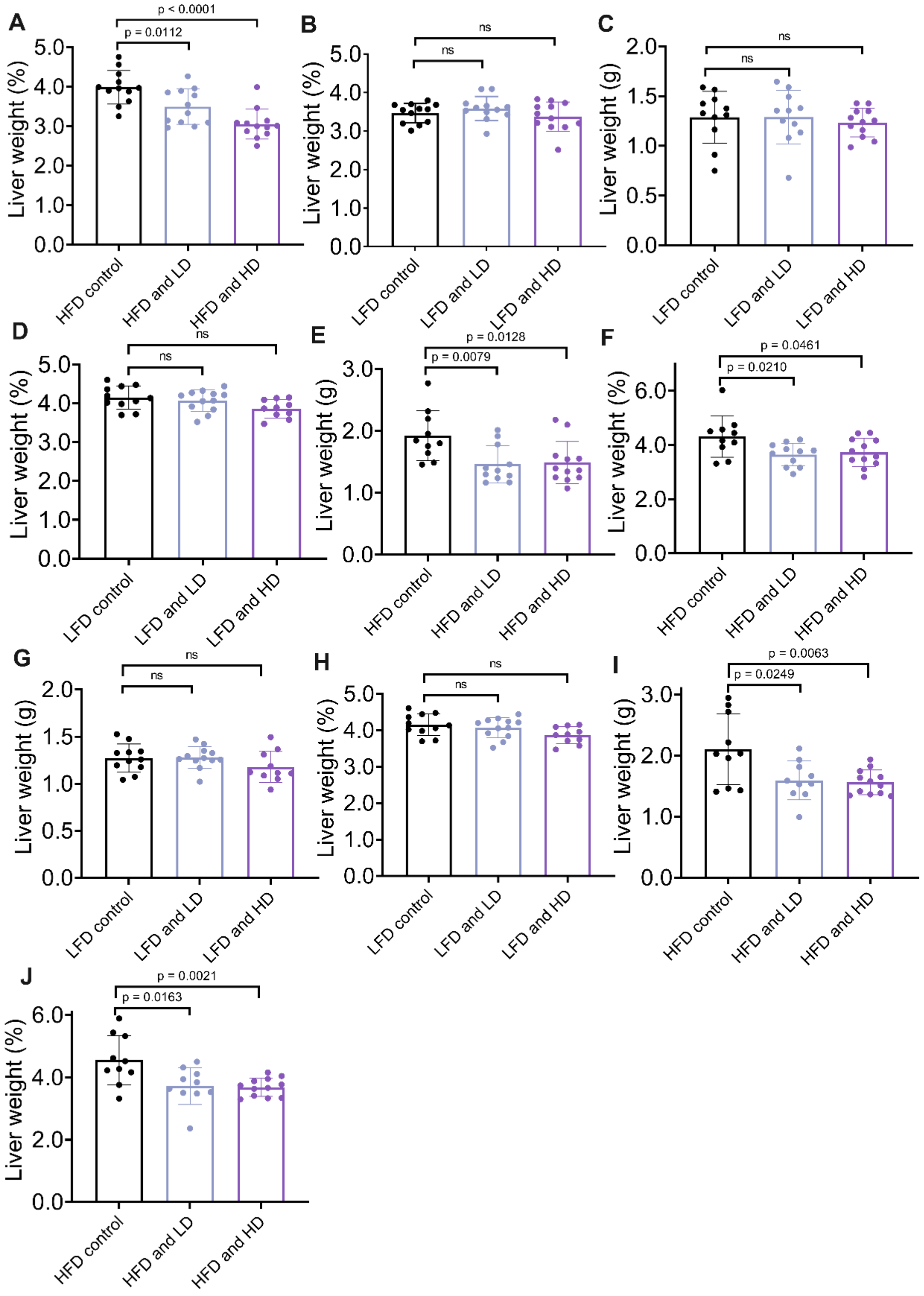

Supplementary Figure. 6.

Liver weight of C57BL/6J DIO mice after multiple doses of acNPs, ipGTT and ipITT. (A) Multiple injections of acNPs to mice result in significant liver weight/body weight reduction, with more significant reductions with high dose acNPs (n=12 animals per condition). (B) Multiple

injections of acNPs to LFD mice do not result in significant liver weight/body weight reduction (n=12 animals per condition). (C – D) Liver weight of LFD mice did not change after ipGTT (For C, n=11 for LFD control, n=11 for LFD and LD, n=12 for LFD and HD; For D, n=11 animals for LFD control, n=12 animals for LFD and LD, n=10 animals for LFD and HD). (E – F) Liver weight of HFD mice maintained the same amount of reduction with acNPs treatment after ipGTT (For E, n=10 animals for HFD control, n=11 animals for HFD and LD, n=12 animals for HFD and HD; For F, n=10 animals for LFD control, n=11 animals for LFD and LD, n=12 animals for LFD and HD. (G – H) Liver weight of LFD mice did not change after ipITT (For G, n=11 animals for LFD control, n=12 animals for LFD and LD, n=10 animals for LFD and HD; For H, n=10 animals for LFD control, n=10 animals for LFD and LD, n=12 animals for LFD and HD). (I – J) Liver weight of HFD mice maintained the same amount of reduction with acNPs treatment after ipITT. For I, (n=10 animals for HFD control, n=10 animals for HFD and LD, n=12 animals for HFD and HD). For J, (n=10 animals for HFD control, n=10 animals for HFD and LD, n=12 animals for HFD and HD). Two-tailed unpaired t test (A – J); data are expressed as means ± SD. n.s. not statistically significant. Source data are provided as a Source Data file.

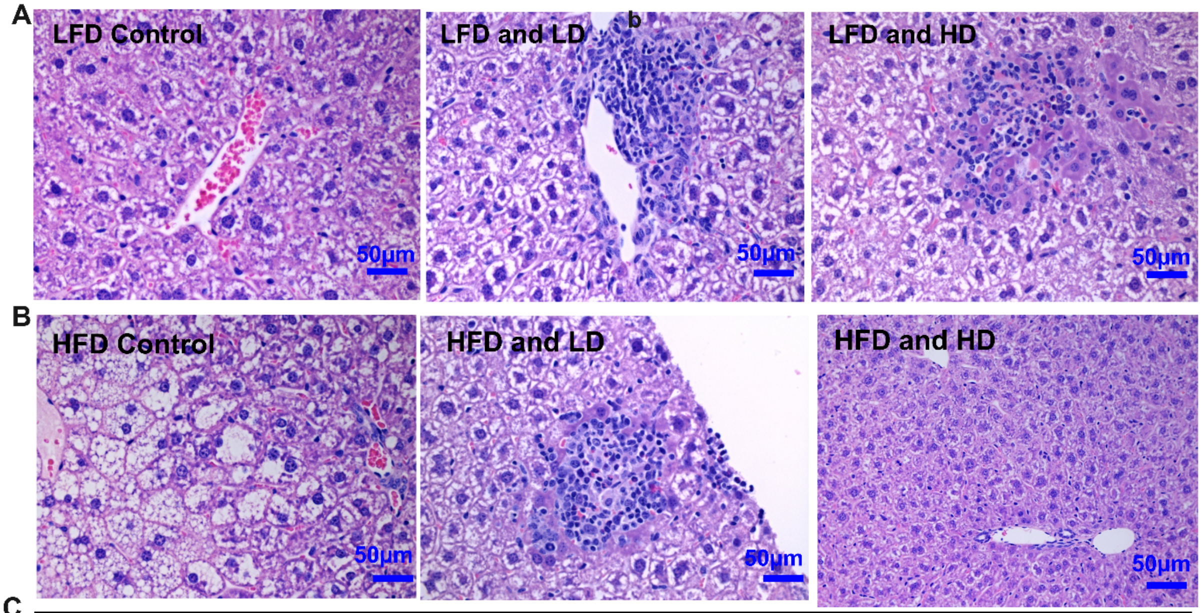

C

Mice type			LFD			HFD		
Injection type			Multiple			Multiple		
Item	Definition	Score	Control	LD	HD	Control	LD	HD
Steatosis (Grade)	<5%	0	**0**	**0**	**0**			
	5%-33%	1						**1**
	>33%-66%	2					**2**	
	>66%	3				**3**		

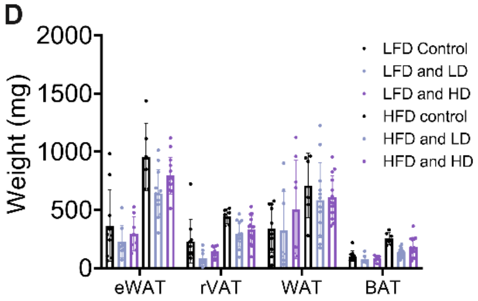

Supplementary Figure. 7.

H & E liver sections of C57BL/6J DIO mice after multiple doses of acNPs, ipGTT and ipITT.

(A – B) Representative H & E stains of LFD and HFD mice liver slices after ipGTT show maintained reduction in steatosis in livers with acNPs treatment. n= 3 animals per treatment condition. (C) Scoring shows lower steatosis for HFD mice. (D) Mice fat pads weight measurements in mice shows that the weight reduction is localized in the liver. (n=10 animals for LFD control, n=8 animals for LFD and LD, n=8 animals for LFD and HD, n=6 animals for HFD control, n=12 animals for HFD and LD, n=14 animals for HFD and HD). Data are expressed as means ± SD. Source data are provided as a Source Data file.

			Day 1			Day 6		
Item	Definition	Score	Control	LD	HD	Control	LD	HD
Steatosis								
Grade	Low- to medium-power evaluation of parenchymal involvement by steatosis							
	<5%	0					**0**	**0**
	5%-33%	1		**1**				
	>33%-66%	2	**2**		**2**	**3**		
	>66%	3						
Location	Predominant distribution pattern							
	Zone 3	0	**0**	**0**	**0**	**0**	**0**	**0**
	Zone 1	1						
	Azonal	2						
	Panacinar	3						
Microvesicular steatosis	Not present	0				**0**		**0**
	Present	1	**1**	**1**	**1**		**1**	
Fibrosis<br>Stage	None	0	**0**	**0**	**0**	**0**	**0**	**0**
	Perisinusoidal or periportal	1						
	Mild, zone 3, perisinusoidal	1A						
	Moderate, zone 3, perisinusoidal	1B						
	Portal/periportal	1C						
	Perisinusoidal and portal/periportal	2						
	Bridging fibrosis	3						
	Cirrhosis	4						
Inflammation<br>Lobular inflammation	No foci	0	**0**	**0**	**0**	**0**	**0**	
	<2 foci per 200× field	1						**1**
	2-4 foci per 200× field	2						
	>4 foci per 200× field	3						
Microgranulomas	Absent	0	**0**	**0**	**0**	**0**	**0**	**0**
	Present	1						
Large lipogranulomas	Absent	0	**0**	**0**	**0**	**0**	**0**	**0**
	Present	1						
Portal inflammation	None to minimal	0	**0**	**0**	**0**	**0**	**0**	**0**
	Greater than minimal	1						
Liver cell injury<br>Ballooning	None	0	**0**	**0**	**0**	**0**	**0**	**0**
	Few balloon cells	1						
	Many cells/prominent ballooning	2						
Acidophil bodies	None to rare	0	**0**	**0**	**0**	**0**	**0**	**0**
	Many	1						
Pigmented macrophages	None to rare	0	**0**	**0**	**0**	**0**	**0**	**0**
	Many	1						
Megamitochondria	None to rare	0	**0**	**0**	**0**	**0**	**0**	**0**
	Many	1						
Mallory's hyaline	None to rare	0	**0**	**0**	**0**	**0**	**0**	**0**
	Many	1						
Glycogenated nuclei	None to rare	0	**0**	**0**	**0**	**0**	**0**	**0**
	Many	1						
Diagnostic classification	Not steatohepatitis	0	**0**	**0**	**0**	**0**	**0**	**0**
	Possible/borderline	1						
	Definite steatohepatits	2						

Supplementary Table 3.

Histopathological scoring grid for NAFLD/NASH liver sections.

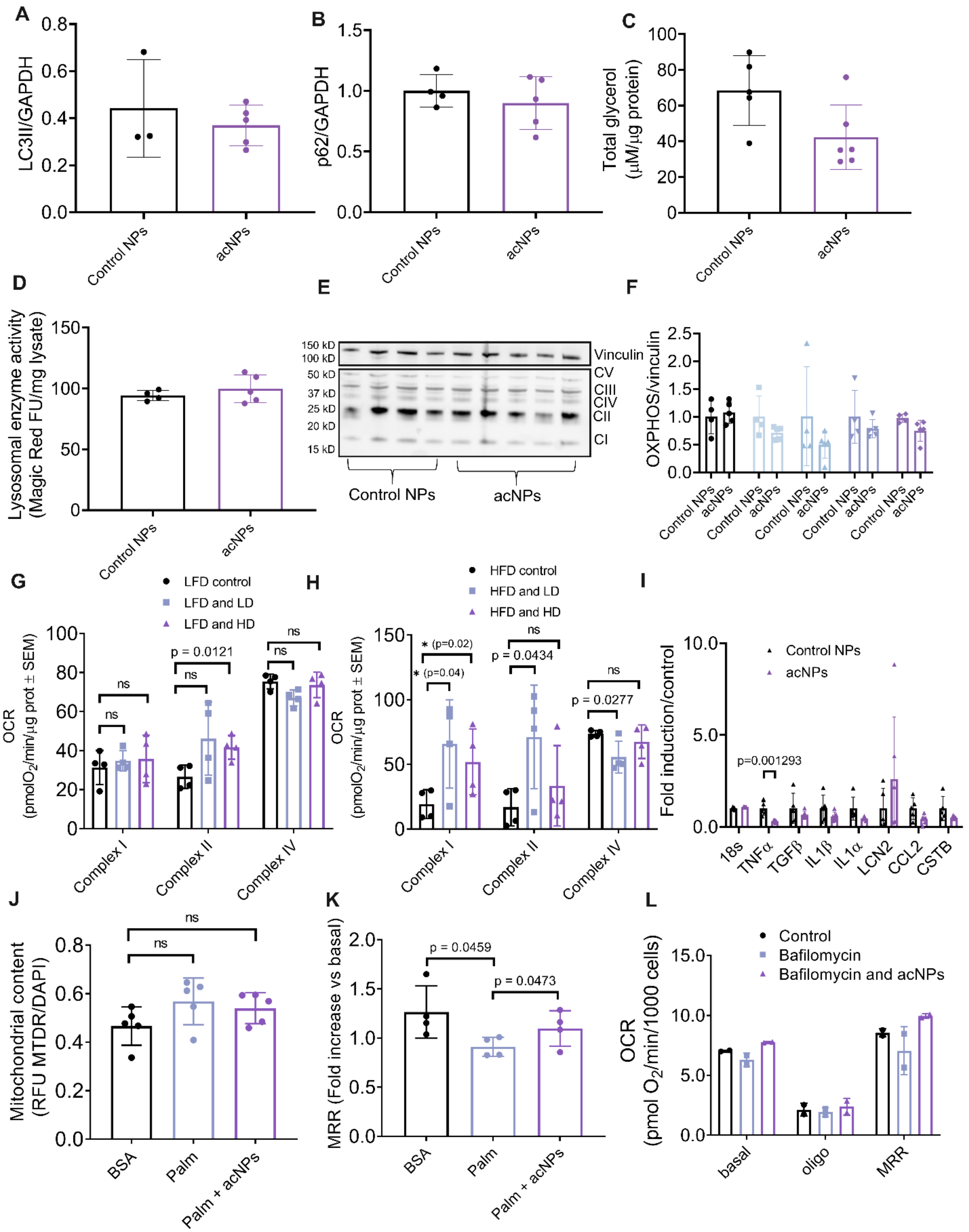
A
LC3II/GAPDH
0.0
0.2
0.4
0.6
0.8
Control NPs
acNPs
B
p62/GAPDH
0.0
0.5
1.0
1.5
Control NPs
acNPs
C
Total glycerol
(μM/μg protein)
0
20
40
60
80
100
Control NPs
acNPs
D
Lysosomal enzyme activity
(Magic Red FU/mg lysate)
0
50
100
150
Control NPs
acNPs
E
150 kD
100 kD
50 kD
37 kD
25 kD
20 kD
15 kD
Vinculin
CV
CIII
CIV
CII
CI
Control NPs
acNPs
F
OXPHOS/vinculin
0.0
0.5
1.0
1.5
2.0
2.5
Control NPs
acNPs
G
LFD control
LFD and LD
LFD and HD
OCR
(pmolO$_2$/min/μg prot ± SEM)
0
20
40
60
80
100
ns
p = 0.0121
Complex I
Complex II
Complex IV
H
HFD control
HFD and LD
HFD and HD
OCR
(pmolO$_2$/min/μg prot ± SEM)
0
50
100
150
* (p=0.02)
* (p=0.04)
ns
p = 0.0434
p = 0.0277
Complex I
Complex II
Complex IV
I
Control NPs
acNPs
Fold induction/control
0.0
5.0
10.0
p=0.001293
18s
TNFα
TGFβ
IL1β
IL1α
LCN2
CCL2
CSTB
J
Mitochondrial content
(RFU MTDR/DAPI)
0.0
0.2
0.4
0.6
0.8
ns
BSA
Palm
Palm + acNPs
K
MRR (Fold increase vs basal)
0.0
0.5
1.0
1.5
2.0
p = 0.0459
p = 0.0473
BSA
Palm
Palm + acNPs
L
Control
Bafilomycin
Bafilomycin and acNPs
OCR
(pmol O$_2$/min/1000 cells)
0.0
5.0
10.0
15.0
basal
oligo
MRR

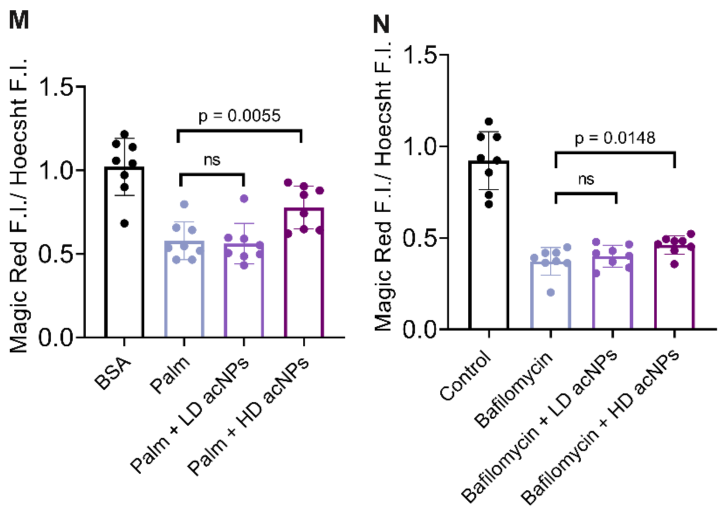

Supplementary Figure. 8.

Liver autophagy function, mitochondria oxidative capacity, lysosomal enzyme activity, as well as liver inflammatory cytokines RNA expression levels in either C57BL/6N mice or C57BL/6J mice. Replication of the acNPs treatment study was done in C57BL/6N mice. (A – B) Quantification of LC3-II and p62 expression data in C57BL/6N mice (n=3 animals for control NPs, n=5 animals for acNPs). (C) acNPs reduce triglyceride levels in C57BL/6N mice livers compared to control NPs (n=5 animals for control NPs, n=6 animals for acNPs). (D) acNPs increase lysosomal enzyme activity compared to control NPs in C57BL/6N mice (n=4 animals for control NPs, n=5 animals for acNPs). (E – F) Western blot and quantification of mitochondrial complex I-V content. acNPs treatment do not significantly change mitochondrial content (n=4 animals for control NPs, n=5 animals for acNPs). (G) Mitochondrial oxygen consumption rate of C57BL/6J frozen mice liver lysates with LFD control, LFD and LD acNPs and LFD and HD acNPs treatment conditions (n=4 animals per treatment group). (H) Mitochondrial oxygen consumption rate of C57BL/6J frozen mice liver lysates with HFD control, HFD and LD acNPs and HFD and HD acNPs treatment conditions (n=4 animals per treatment group). (I) Liver inflammatory cytokines RNA expression levels. acNPs treatment reduce most cytokine levels, with TNF-α being most significant (n=5 animals for control NPs, n=6 animals for acNPs). (J) Mitochondria content (n=5 animals per treatment condition) and (K) Maximal respiratory rate of different treatment conditions on primary human hepatocytes. (n=4 animals per treatment condition). (L) Mitochondrial oxygen consumption rate of primary human hepatocytes under different treatment conditions (N=2 independent experiments). (M – N) Magic red activity of LD and HD acNPs treated primary human hepatocytes, n=8 animals per treatment condition. Oligo: Oligomycin, MRR: Maximal respiratory rate. Two-tailed unpaired t test (G, H, I, J, K, M, N); data are expressed as means ± SD. n.s. not statistically significant. Source data are provided as a Source Data file.

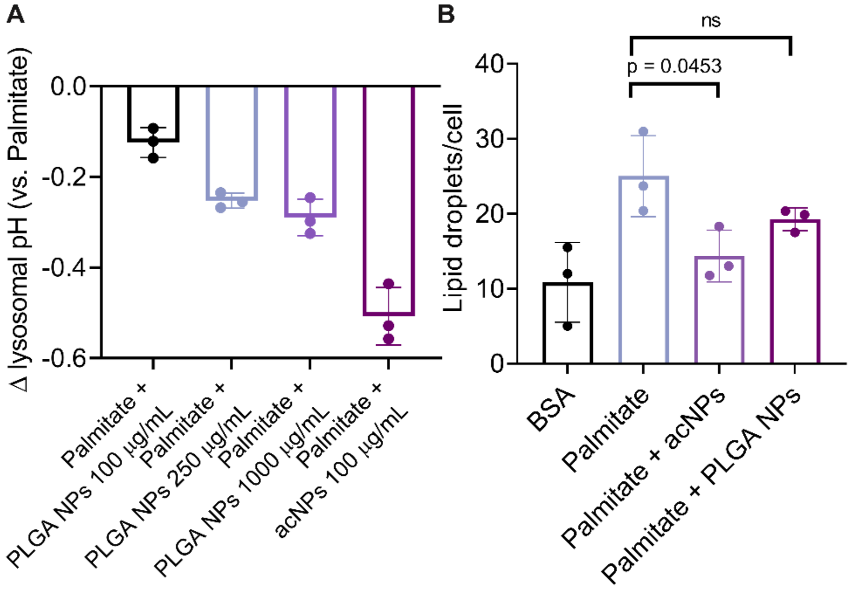

Supplementary Figure. 9.

Comparison between acNPs and PLGA NPs in HepG2 cells. (A) Quantification of lysosomal pH in palmitate treated HepG2 cells with either acNPs or PLGA NPs (N=3 independent experiments). (B) Quantification of HepG2 lipid droplets with either acNPs or PLGA NPs using Nile red staining (N=3 independent experiments). Two-tailed unpaired t test (B); data are expressed as means ± SD. n.s. not statistically significant. Source data are provided as a Source Data file.

Known equilibrium and Ka values:

$$K_{1,TFSA} = \frac{[H^+][M_1^-]}{[M_1H]} = 2.51 * 10^{-2} \text{ (1)}$$

$$K_{2,SA} = \frac{[H^+][M_2^-]}{[M_2H]} = 6.31 * 10^{-5} \text{ (2)}$$

Upon addition of NPs to solution, we assume that all generated acid in solution is due to polymer degradation

$$[H^+] = [H^+]_{pH\,7,\ t=0h} + [M_1^-] + [M_2^-] \text{ (3)}$$

$$[M_2^-] = [H^+] - [H^+]_{pH\,7,\ t=0h} - [M_1^-] \text{ (4)}$$

$$[M_1^-] = [H^+] - [H^+]_{pH\,7,\ t=0h} - [M_2^-] \text{ (5)}$$

We assume the acids can be either protonated or unprotonated in solution and that the acids in solution are equivalent to the monomer ratios within the polymer:

$$[TFSA] = [M_1H] + [M_1^-] \qquad [SA] = [M_2H] + [M_2^-] \text{ (6)}$$

$$[TFSA] = 0.25[Total] \qquad [SA] = 0.75[Total] \text{ (7)}$$

$$[Total] = [TFSA] + [SA] \text{ (8)}$$

We then use the two separate equations defining the total amount of monomer, [Total], in solution equal to one another, substitute, and solve for $[M_1^-]$

$$[Total] = 4[TFSA] = 4([M_1H] + [M_1^-]) = 4(\frac{[H^+][M_1^-]}{[k_1]} + [M_1^-]) \text{ (9)}$$

$$[Total] = [M_1H] + [M_1^-] + [M_2H] + [M_2^-] = \frac{[H^+][M_1^-]}{k_1} + [M_1^-] + \frac{[H^+]([H^+] - [H^+]_{pH\,7} - [M_1^-])}{k_2} + [H^+] - [H^+]_{pH\,7} \text{ (10)}$$

$$[M_1^-] = \frac{\frac{[H^+]^2}{k_2} - \frac{[H^+][H^+]_{pH\,7}}{k_2} + [H^+] - [H^+]_{pH\,7}}{\frac{3[H^+]}{k_1} + 4 + \frac{[H^+]}{k_2}} \text{ (11)}$$

Supplementary Note. Calculation of the amount of tetrafluorosuccinic acid (TFSA) or succinic acid (SA) required to generate the pH changes as measured in pH 6.0 buffer.

Additional materials and methods

**TNF-α**				
PrimerBank ID	133892368c3			
Amplicon Size	148			
	Sequence (5' → 3')	Length	T_m	Location
Forward Primer	CCTGTAGCCCACGTCGTAG	19	61.5	271-289
Reverse Primer	GGGAGTAGACAAGGTACAACCC	22	61.4	418-397
**TGF-β**				
PrimerBank ID	6755774c1			
Amplicon Size	91			
	Sequence (5' → 3')	Length	T_m	Location
Forward Primer	CCACCTGCAAGACCATCGAC	20	62.8	92-111
Reverse Primer	CTGGCGAGCCTTAGTTTGGAC	21	63	182-162
**IL-1α**				
PrimerBank ID	118130060c1			
Amplicon Size	109			
	Sequence (5' → 3')	Length	T_m	Location
Forward Primer	TCTATGATGCAAGCTATGGCTCA	23	61.3	104-126
Reverse Primer	CGGCTCTCCTTGAAGGTGA	19	61	212-194
**IL-1β**				
PrimerBank ID	118130747c1			
Amplicon Size	116			
	Sequence (5' → 3')	Length	T_m	Location
Forward Primer	GAAATGCCACCTTTTGACAGTG	22	60.2	28-49
Reverse Primer	TGGATGCTCTCATCAGGACAG	21	61	142-123
**LCN2**				
PrimerBank ID	1019908a1			
Amplicon Size	239			
	Sequence (5' → 3')	Length	T_m	Location
Forward Primer	TGGCCCTGAGTGTCATGTG	19	61.6	2-20
Reverse Primer	CTCTTGTAGCTCATAGATGGTGC	23	60.2	240-218
**CCL2**				
PrimerBank ID	6755430a1			
Amplicon Size	121			
	Sequence (5' → 3')	Length	T_m	Location
Forward Primer	TTAAAAACCTGGATCGGAACCAA	23	60.1	260-282
Reverse Primer	GCATTAGCTTCAGATTTACGGGT	23	60.7	380-358
**CSTB**				
PrimerBank ID	6681071a1			
Amplicon Size	196			
	Sequence (5' → 3')	Length	T_m	Location
Forward Primer	AGGTGAAGTCCCAGCTTGAAT	21	61.1	65-85
Reverse Primer	GTCTGATAGGAAGACAGGGTCA	22	60.3	260-239
**18S rRNA**				
PrimerBank ID	6755368a1			
Amplicon Size	166			
	Sequence (5' → 3')	Length	T_m	Location
Forward Primer	AGTTCCAGCACATTTTGCGAG	21	61.4	23-43
Reverse Primer	TCATCCTCCGTGAGTTCTCCA	21	62.1	188-168
