# Supplementary material for: Restoration of lysosomal acidification rescues autophagy and metabolic dysfunction in non-alcoholic fatty liver disease
Source: Nat Commun. 2023 May 4;14:2573. doi: 10.1038/s41467-023-38165-6 (PMC10160018; doi:10.1038/s41467-023-38165-6)
Supplement: Supplementary file 3 — Reporting Summary [file 41467_2023_38165_MOESM3_ESM.pdf]

nature portfolio

Corresponding author(s): Mark Grinstaff

Last updated by author(s): Mar 11, 2023

# Reporting Summary

Nature Portfolio wishes to improve the reproducibility of the work that we publish. This form provides structure for consistency and transparency in reporting. For further information on Nature Portfolio policies, see our Editorial Policies and the Editorial Policy Checklist.

## Statistics

For all statistical analyses, confirm that the following items are present in the figure legend, table legend, main text, or Methods section.

| n/a | Confirmed | |
|---|---|---|
| ☐ | ☒ | The exact sample size (*n*) for each experimental group/condition, given as a discrete number and unit of measurement |
| ☐ | ☒ | A statement on whether measurements were taken from distinct samples or whether the same sample was measured repeatedly |
| ☐ | ☒ | The statistical test(s) used AND whether they are one- or two-sided<br>*Only common tests should be described solely by name; describe more complex techniques in the Methods section.* |
| ☒ | ☐ | A description of all covariates tested |
| ☒ | ☐ | A description of any assumptions or corrections, such as tests of normality and adjustment for multiple comparisons |
| ☐ | ☒ | A full description of the statistical parameters including central tendency (e.g. means) or other basic estimates (e.g. regression coefficient) AND variation (e.g. standard deviation) or associated estimates of uncertainty (e.g. confidence intervals) |
| ☐ | ☒ | For null hypothesis testing, the test statistic (e.g. *F*, *t*, *r*) with confidence intervals, effect sizes, degrees of freedom and *P* value noted<br>*Give P values as exact values whenever suitable.* |
| ☒ | ☐ | For Bayesian analysis, information on the choice of priors and Markov chain Monte Carlo settings |
| ☒ | ☐ | For hierarchical and complex designs, identification of the appropriate level for tests and full reporting of outcomes |
| ☒ | ☐ | Estimates of effect sizes (e.g. Cohen's *d*, Pearson's *r*), indicating how they were calculated |

*Our web collection on statistics for biologists contains articles on many of the points above.*

## Software and code

Policy information about availability of computer code

| | |
|---|---|
| Data collection | Zeiss LSM 880 Confocal Microscope Metamorph imaging software, Zeiss Supra 55VP field emission scanning electron microscope, Varian INOVA 500MHz spectrometer, Gel permeation chromatography, Brookehaven dynamic light scattering instrument. |
| Data analysis | Tecan 2000 plate reader, Wave software (Agilent), Gel Doc XR+ Gel Documentation System (Image Lab software), Fiji Image J v.1.5.3s, Biotek Synergy HT plate reader, Microsoft Excel, QuPath v.0.2.3, ORIGIN PRO 8. All statistical analysis were performed in GraphPad Prism 9. |

For manuscripts utilizing custom algorithms or software that are central to the research but not yet described in published literature, software must be made available to editors and reviewers. We strongly encourage code deposition in a community repository (e.g. GitHub). See the Nature Portfolio guidelines for submitting code & software for further information.

## Data

Policy information about availability of data

All manuscripts must include a data availability statement. This statement should provide the following information, where applicable:

- Accession codes, unique identifiers, or web links for publicly available datasets
- A description of any restrictions on data availability
- For clinical datasets or third party data, please ensure that the statement adheres to our policy

The authors declare that source data are provided with this paper. The PrimerBank IDs for primers used for qPCR analysis can be found in the supplementary information file. Additionally, requests for materials can be also addressed to Professor Mark Grinstaff (mgrin@bu.edu).

## Human research participants

Policy information about studies involving human research participants and Sex and Gender in Research.

| | |
|---|---|
| Reporting on sex and gender | *Use the terms sex (biological attribute) and gender (shaped by social and cultural circumstances) carefully in order to avoid confusing both terms. Indicate if findings apply to only one sex or gender; describe whether sex and gender were considered in study design whether sex and/or gender was determined based on self-reporting or assigned and methods used. Provide in the source data disaggregated sex and gender data where this information has been collected, and consent has been obtained for sharing of individual-level data; provide overall numbers in this Reporting Summary. Please state if this information has not been collected. Report sex- and gender-based analyses where performed, justify reasons for lack of sex- and gender-based analysis.* |
| Population characteristics | *Describe the covariate-relevant population characteristics of the human research participants (e.g. age, genotypic information, past and current diagnosis and treatment categories). If you filled out the behavioural & social sciences study design questions and have nothing to add here, write "See above."* |
| Recruitment | *Describe how participants were recruited. Outline any potential self-selection bias or other biases that may be present and how these are likely to impact results.* |
| Ethics oversight | *Identify the organization(s) that approved the study protocol.* |

Note that full information on the approval of the study protocol must also be provided in the manuscript.

# Field-specific reporting

Please select the one below that is the best fit for your research. If you are not sure, read the appropriate sections before making your selection.

☒ Life sciences ☐ Behavioural & social sciences ☐ Ecological, evolutionary & environmental sciences

For a reference copy of the document with all sections, see nature.com/documents/nr-reporting-summary-flat.pdf

# Life sciences study design

All studies must disclose on these points even when the disclosure is negative.

| | |
|---|---|
| Sample size | The sample size was chosen based on prior experiences with the techniques, and other references performing similar studies. Per IACUC compliance, we used the minimal sample size that would give statistical significant differences for the parameters measured. Sample sizes for each assay or animal study has been included in the figure legends. |
| Data exclusions | Exclusion criteria include presence, after veterinary assessment, of infections, ulcerative dermatitis, bladder enlargements or other common diseases in the mouse strains used. |
| Replication | The experimental findings were reproduced and all replication attempts were successful. Experiments were replicated or performed independently at least one week apart from the each other. |
| Randomization | The strains of different mice were made known during the animal study. In each animal experiment, mice were randomly allocated to receive tail vein injections of either saline injection or with low dose or high dose acNPs. |
| Blinding | Personnel handling the animals were blinded to the different experimental groups when conducting glucose and insulin tolerance tests. For the histology sections on mice liver samples, samples were assigned a code number, and the identification of samples and analysis/ determination of the degree of steatosis were done by different people. The other studies were not blinded. |

# Reporting for specific materials, systems and methods

We require information from authors about some types of materials, experimental systems and methods used in many studies. Here, indicate whether each material, system or method listed is relevant to your study. If you are not sure if a list item applies to your research, read the appropriate section before selecting a response.

## Materials & experimental systems

| n/a | Involved in the study |
|---|---|
| ☐ | ☒ Antibodies |
| ☐ | ☒ Eukaryotic cell lines |
| ☒ | ☐ Palaeontology and archaeology |
| ☐ | ☒ Animals and other organisms |
| ☒ | ☐ Clinical data |
| ☒ | ☐ Dual use research of concern |

## Methods

| n/a | Involved in the study |
|---|---|
| ☒ | ☐ ChIP-seq |
| ☐ | ☒ Flow cytometry |
| ☒ | ☐ MRI-based neuroimaging |

# Antibodies

| | |
|---|---|
| Antibodies used | LC3A/B (Cell Signaling, Cat # 12741; RRID: AB_2617131, 1:1000), GAPDH (Cell Signaling, Cat # 2118; RRID: AB_561053, 1:1000), β-actin (Cell Signaling, Cat # 4967; RRID: AB_330288, 1:1000), SQSTM1/p62 (Cell Signaling, Cat # 5114; RRID: AB_10624872, 1:1000), Akt (Cell Signaling, Cat # 4685; RRID: AB_2225340, 1:1000), p-Akt (Ser 473) (Cell Signaling, Cat # 4060; RRID: AB_2315049, 1:1000), Insulin Receptor β (IR) (Cell Signaling, Cat # 3025; RRID: AB_2280448, 1:1000), p-IR Tyr1162/1163 (Invitrogen, Cat # 44-84; RRID: AB_2533762, 1:1000), GSK-3β (Cell Signaling, Cat # 12456; RRID: AB_2636978, 1:1000), p-GSK3β (Ser 9) (Cell Signaling, Cat # 9336; RRID: AB_331405, 1:1000), LAMP-1 (BioLegend, Cat # 121602; RRID: AB_572021), anti-CLEC4F (BioLegend, Cat # 156803; RRID: AB_2814081), Total OXPHOS Rodent WB Antibody Cocktail (Abcam, Cat # ab110413; RRID: AB_2629281, 1:1000), Vinculin (Sigma Aldrich, Cat # V9131; RRID: AB_477629, 1:1000), Anti-rabbit IgG, HRP-linked Antibody (Cell Signaling, Cat # 7074; RRID: AB_2099233, 1:3000), Anti-mouse IgG, HRP-linked Antibody (Cell Signaling, Cat # 7076; RRID: AB_330924; 1:3000), Anti-Mouse Alexa Fluor 488 (Thermofisher Scientific, Cat # A11001; RRID: AB_2534069) |
| Validation | Abcam statement on validation: "We use application-specific criteria to pass or fail our antibodies and typically test all of our antibodies in multiple applications. These criteria include basic standards, like a specific signal and relevant controls."<br>Cell signaling statement on validation: "CST™ antibodies are produced in-house and validated extensively according to a rigorous protocol."<br>BioLegend statement on validation: "To ensure they are both specific and sensitive, we validate our antibodies through a variety of methods including: Testing on multiple cell and tissue types with a variety of known expression levels."<br>Invitrogen statement on validation: "Invitrogen antibodies are currently undergoing a rigorous two-part testing approach: Part 1—Target specificity verification and Part 2—Functional application validation." |

# Eukaryotic cell lines

Policy information about cell lines and Sex and Gender in Research

| | |
|---|---|
| Cell line source(s) | HepG2 cells and primary human hepatocytes were obtained from ATCC. |
| Authentication | No authentication of the cell lines was performed. |
| Mycoplasma contamination | Cells were used as bought from the vendor. |
| Commonly misidentified lines (See ICLAC register) | No commonly misidentified lines were used in this study. |

# Animals and other research organisms

Policy information about studies involving animals; ARRIVE guidelines recommended for reporting animal research, and Sex and Gender in Research

| | |
|---|---|
| Laboratory animals | C57BL/6J DIO control (male 16 weeks) and C57BL/6J DIO control (male 16 weeks), FVB/NJ and C57BL/6N (male, 17 weeks) were used in this study. The mice were housed in a temperature-controlled room (25°C) in virus-free facilities on a 12-h light/dark cycle (7:30 a.m. on/7:30 p.m. off) and water ad libitum. Experimental procedures conducted on mice were performed in accordance with animal welfare and in compliance with other related ethical regulations. The mice studies were conducted under approved Institutional Animal Care and Use Committee (IACUC) protocols at both Boston University and University of California, Los Angeles. |
| Wild animals | This study did not involve wild animals. |
| Reporting on sex | We only chose male mice because female mice have been reported to be protected against high fat diet induced metabolic syndrome. |
| Field-collected samples | This study did not involve samples collected from the field study. |
| Ethics oversight | The animal protocol was approved by Boston University, with protocol number 17-014, and by the University of California, Los Angeles, with protocol number ARC-2017-019. |

Note that full information on the approval of the study protocol must also be provided in the manuscript.

# Flow Cytometry

## Plots

Confirm that:

- [x] The axis labels state the marker and fluorochrome used (e.g. CD4-FITC).
- [x] The axis scales are clearly visible. Include numbers along axes only for bottom left plot of group (a 'group' is an analysis of identical markers).
- [x] All plots are contour plots with outliers or pseudocolor plots.
- [x] A numerical value for number of cells or percentage (with statistics) is provided.

## Methodology

| | |
|---|---|
| Sample preparation | FACS analyses of rhodamine-labelled acNP-treated cells was done with 620 FACScan. FACS data analysis was performed using FACScalibur (Beckman Coulter). 500,000 cells per timepoint were trypsinized, washed twice with PBS by centrifugation, and then subjected to flow cytometry. Cell debris was excluded by gating on the forward and side scatter plot. |
| Instrument | BD 620 FACScan |
| Software | FACScalibur (Beckman Coulter) |
| Cell population abundance | 500, 000 cells per sample |
| Gating strategy | Gating strategy is applied on the FSC/SSC gates to indicate the population of viable cells. The fluorescence intensity cut-off is then applied on the FL3-H (red fluorescence) channel to indicate the amount of viable cells that have uptaken rhodamine-labelled nanoparticles. |

- [x] Tick this box to confirm that a figure exemplifying the gating strategy is provided in the Supplementary Information.
